# Combining microfluidic paper-based platform and metal–organic frameworks in a single device for phenolic content assessment in fruits

**DOI:** 10.1007/s00604-023-05702-5

**Published:** 2023-03-10

**Authors:** H. Martínez-Pérez-Cejuela, Raquel B. R. Mesquita, E. F. Simó-Alfonso, J. M. Herrero-Martínez, António O. S. S. Rangel

**Affiliations:** 1 Department of Analytical Chemistry, University of Valencia, Dr Moliner 50, 46100 Burjassot, Valencia Spain; 2 Universidade Católica Portuguesa, CBQF - Centro de Biotecnologia e Química Fina - Laboratório Associado, Escola Superior de Biotecnologia, Rua Diogo Botelho 1327, 4169-005 Porto, Portugal

**Keywords:** Folin Index, Fruit sample analysis, Portable device, Preconcentration, ZIF-8, Colorimetry, Digital imaging

## Abstract

A microfluidic paper-based device (µPAD) has been combined with metal–organic frameworks (MOFs) for total phenolic compounds (TPC) quantification in fruit samples for the first time. The performance of the µPAD, based upon the vertical flow approach, was enhanced in order to determine the TPC content with high accuracy in fruit samples. The method was based on the traditional Folin-Ciocalteu Index using gallic acid or oenotannin as reference phenolic compounds. This novel design and construction of the device are in agreement with the principles of Green Chemistry avoiding wax technology (lower toxicity). The analytical parameters that affect the colorimetric method (using digital imaging of the colored zone) performance were optimized including design, sample volume, and MOF amount. Then, the analytical features of the developed method were investigated such as dynamic range (1.6–30 mg L^−1^), limit of detection (0.5 mg L^−1^), and precision (RSD < 9%). Besides, the in-field analysis is achievable with a color stability up to 6 h after the loading process of the sample and storage stability for at least 15 days without performance losses (under vacuum at − 20 °C). Furthermore, the MOF ZIF-8@paper was characterized to study its composition and the successful combination. The feasibility of the proposed method was demonstrated by determining the TPC in 5 fruit samples using oenotannin as reference solute. The accuracy was validated by comparison of the data with the results obtained with the recommended protocol proposed by the International Organisation of Vine and Wine (OIV).

**Graphical Abstract:**

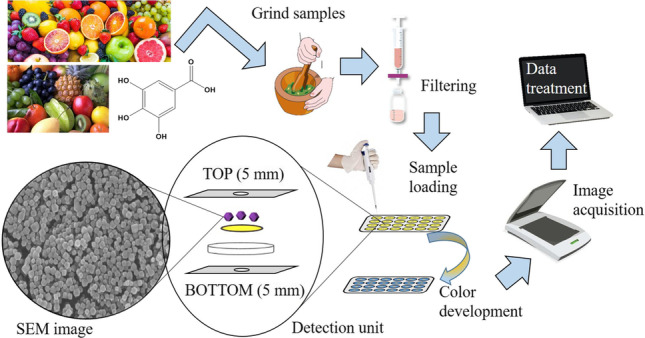

**Supplementary Information:**

The online version contains supplementary material available at 10.1007/s00604-023-05702-5.

## Introduction

In the past decade, the effectiveness and efficiency of methods have been the most sought-after analytical features. However, that preference has recently moved to other desirable features such as miniaturization and sustainability due to the awareness of the importance of researching the impact on the environment. In this context, microfluidic paper-based analytical devices (μPADs) have emerged as an alternative to traditional methods in analytical chemistry owing to their advantages including cost-effectiveness, user-friendliness, portability, and so on [1, 2]. Furthermore, the cellulose nature (porosity and hydrophilicity) allows the flow generation via capillary forces without any further external force [3]. Due to these characteristics, which are in accordance with the principles of Green Analytical Chemistry [4], many efforts have been made in the development of paper-based methods becoming a powerful tool not only for the medical point-of-care diagnosis [5], but also in other important applications such as environmental monitoring [6], heavy metals detection [7], and food control [8].

Regarding the µPAD design, we encourage the reader to consult recent excellent reviews for further insight [1, 2]. Briefly, there are two clearly defined regions, the hydrophilic (cellulosic substrate) and the hydrophobic area (delimits the reaction zone). They can be constructed using different techniques such as wax printing, inkjet technology, among others [9], all of them compatible with most reported detection modes (electrochemistry, conductivity, fluorescence, and colorimetry) [10]. Despite the good suitability of µPAD for remote analysis, there are still some drawbacks to be addressed. For instance, the low sensitivity during the colorimetric probes [10] or the low amount of absorbed analytes as a consequence of the use of limited volumes [11], among others. In this context, the hybridization with reticular materials could be a good alternative to overcome these problems and to meet the demands of the new analytical challenges, especially, trace levels of analytes in complex sample matrices.

Within the reticular materials, metal–organic frameworks (MOFs) are crystalline porous materials based on organic ligands and inorganic ion precursors, which present outstanding features (e.g., easy functionalization, exceptional porosity, facile characterization, and so forth) [12]. In general, MOFs have been commonly synthesized under solvothermal conditions. However, greener alternatives are recently gaining popularity in order to avoid high pressures and temperatures, neither toxic solvents (e.g., N,N-dimethylformamide, the most common solvent) [13]. Up to now, there are approximately 100,000 reported structures of these structures [14] in a wide range of areas including catalysis, energy storage, food, and environmental safety [12]. Interestingly, their application in analytical chemistry with sample treatment purposes has become an extended topic [15] due to some particular characteristics, which favor the extraction processes, such as large surface area and good stability in different conditions (pH, temperature, organic solvents, harassing conditions).

Thus, the combination of paper substrate with MOFs could be a promising alternative to build a composite taking profit from the permeability and simplicity of the cellulosic paper and capturing the performance of the MOFs [16, 17]. However, only a few works have been published combining those technologies in a single device and the total phenolic compounds (TPC) determination has scarcely been explored. Highlighting five of them, addressed to the food field, there are only two articles using portable tools based on chemiluminescence [18, 19] and none using colorimetric approaches, despite their benefits and the simplicity/advantages of these methods, especially for the colorimetric alternative. These analytes are a heterogeneous group mainly composed of flavonoids, PC, and tannins, which are related to anti-oxidant, anti-aging, and anti-inflammatory properties [20, 21]. Moreover, the PC profile is distinctive for each fruit and even depends on the maturity stage [20, 22]. Thus, its monitoring can help in terms of (i) the establishment of a correlation between consumption-prevention of diseases; (ii) classify different varieties of fruits; and (iii) to know the fruit maturity and to help the farmer to optimize the harvest season.

Bearing in mind all of the previous premises, in this work, we have investigated the hybridization of ZIF-8 with a µPAD technique for TPC quantification in complex fruit samples using the Folin-Ciocalteu (FC) reaction [23]. In so doing, the typical problems with µPADs methods were faced up with the introduction of MOF in the final device. The card design was based on 2 aligned layers with FC reagent and ZIF-8 in the first one and Na_2_CO_3_ in the second one to achieve alkali conditions during the reaction. The method was optimized in terms of design, sample volume, and MOF amount. Furthermore, the ZIF-8@paper was characterized in detail in order to check the correct hybridization between the individual components including studies of Fourier-transform infrared (FT-IR), powder X-ray diffraction (p-XRD), and scanning electron microscopy (SEM), among others. Afterward, the developed method was successfully applied to TPC quantification in several fruits (grapes, pear, apple, blueberry, and strawberry) with different content of PC, comparing the results with the conventional spectrophotometric method as reference values.

## Experimental

### Reagents and solutions

The preparation of solutions along this work was done with Milli-Q water (MQW, resistivity > 18 MΩ cm, Millipore, Bedford, MA, USA) and all the chemicals were analytical grade, unless otherwise stated.

The Folin-Ciocalteu (FC) assays were performed using FC reagent and anhydrous sodium carbonate. The FC reagent was prepared by diluting a commercial FC solution (2 M with respect to the acid, Sigma-Aldrich) to a concentration of 0.5 M with MQW.

The alkali solution of 15% (m/v) sodium carbonate was weekly prepared by weighing anhydrous Na_2_CO_3_ (≥ 98%, Sigma-Aldrich) to a 20 mL MQW.

The methanolic (HPLC grade) ZIF-8 dispersion (10 g L^−1^) was prepared by weighing 20 mg of previously sieved MOF (50–100 µm) and then adjusting the volume to 2 mL with pure methanol (MeOH).

A 1 g L^−1^ stock solution of gallic acid (GA, ≥ 99%, Sigma-Aldrich) was prepared by dissolving 50 mg of the solid in 50 mL MQW and stored at 4 °C in the darkness until used. Daily dilutions were carried out with MQW to prepare working standards in the range of 1–50 mg L^−1^.

The same procedure was followed to perform the oenotannin assays (Martin Vialatte, France) in the analysis of fruit samples.

### Preparation of* ZIF-8*

The preparation of ZIF-8 nanocrystals was done according to previous studies with minor modifications [24, 25]. Briefly, two methanolic solutions were prepared: solution (1) composed of the metal ion Zn (NO_3_)_2_·6H_2_O (1.470 g, 98%) in MeOH (100 mL); and solution (2) of the organic ligand 2-methylimidazole (HMIM, 3.245 g, 99%) in MeOH (100 mL). The molar ratio Zn(II):HMIM:MeOH is approximately 1:8:5000. At room temperature, the solution (2) was dropwise added to (1) under vigorous stirring and a white dispersion was immediately formed. Then, the agitation was kept for 1 day and the solid was separated by centrifugation at 6000 rpm for 15 min. Next, the resulting solid was washed with MeOH (25 mL) three times to remove the unreacted MOF precursors and dried at 50 °C overnight. After that process, the material was ground with a mortar and then sieved with a steel sieve with sizes 50 <  ×  < 100 μm.

### Assembly of µPAD card

**Fig. 1:**
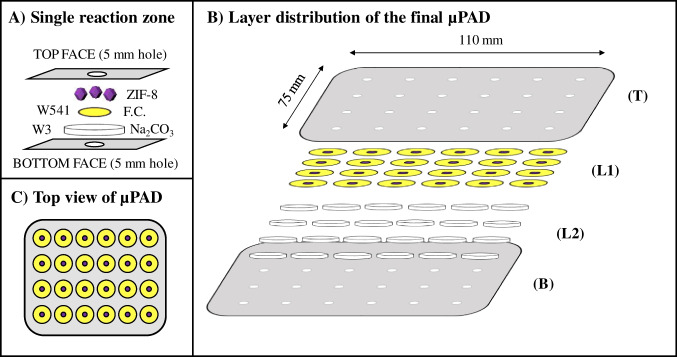
Schematic representation of the final ZIF-8@paper µPAD assembly. **A**, Layer arrangement of a single reaction unit; W541, 6.3 mm Whatman 541 paper disc with 5 µL of 0.5 M Folin-Ciocalteu (FC) and 3 × 5 µL of 10 g L^−1^ ZIF-8; W3, 9.5 mm Whatman 3 paper disc with 20 µL if Na_2_CO_3_ 15% (m/v); **B**, μPAD layer assembly prior to the sealing process; L1, top paper layer, L2 bottom paper layer; T, Top face plastic pouch sheet; B, bottom face plastic pouch sheet; **C**, and top view of a µPAD card after its construction

The basic assembly of the developed device was based on two hydrophilic layers with the FC reagent layer on top (Fig. 1). The µPAD card was based on a vertical flow approach in a 3D design, where the two hydrophilic layers were assembled in 24 determination units separated by the plastic laminating pouch (75 × 110 × 0.125 mm, Q-Connect, Gent, Belgium) as a hydrophobic barrier. Each unit consisted of staking two Whatman® filter paper (Table S1) discs and aligning them between the sampling holes of the laminating pouch (Fig. 1A). After the alignment of the two hydrophilic layers (L1-2, Fig. 1B), the lamination process created the hydrophobic barrier and the top and bottom layers (T-B, Fig. 1B). The FC reagent layer was prepared using a 6.3 mm hardened ashless filter paper (Whatman® W541) cut with stationery punchers (1/4″ EK tools, Lindon, USA) and loaded with 5 µL of 0.5 M FC reagent. Then, the solvent was evaporated (7–8 min in the oven at 50 °C) and the ZIF-8 MOF was deposited by loading 5 µL of 10 g L^−1^ ZIF-8 dispersion followed by evaporation, this step was performed in triplicate. This dispersion was vigorously stirred during the entire process using a magnetic plate in order to assure homogeneity. The second hydrophilic layer (L2 Fig. 1B) was prepared with a 9.5 mm qualitative filter (Whatman® W3) cut with a stationery puncher (3/8″ EK tools, Lindon, USA), loaded with 20 µL of alkali solution and put in the oven for 20 min (at 50 °C).

Then the 24 units were aligned, in a 4 × 6 distribution, with the 5 mm holes (Knipex puncher, Wuppertal, Germany) of the laminating pouch top and bottom sheets (Fig. 1A). After that, the µPAD was sealed with a laminator (United Office, Cleveland, USA) in a unique layer and the device was ready to be used for TPC quantification either immediately or after its storage (Fig. 1C).

In the assembly process, in order to load the MOF onto the reaction zone (W541) with the already dried FC reagent, the dispersion was magnetically stirred to avoid deposition processes (heterogeneity) since it was used a high amount of ZIF-8 particles, even using MeOH (the best dispersion solvent for these particles).

### Characterization of ZIF-8@paper

The spectra of attenuated total reflection FT-IR were registered by putting the paper discs in a DuraSamplIR II accessory from Smiths Detection Inc. (Warrington, UK), which has a nine reflection diamond/ZnSe DuraDisk plates connected on a Bruker FT-IR spectrometer (Bremen, Germany) model Tensor 27.p-XRD patterns were recorded in a D8 Advance A25 diffractometer (Bruker Daltonik GmbH, Bremen, Germany) with the following experimental conditions: Bragg–Brentano geometry, 40 kV, and 40 mA each time. The single diffractogram is the average of five repeated measurements acquired at room temperature from 2θ = 3° to 2θ = 60°.

SEM micrographs, energy dispersive X-ray spectroscopy (EDX) and mapping analysis were obtained with a Hitachi electron microscope (S-4800, Ibaraki, Japan) coupled to a retro-dispersive electron detector and an energy dispersive spectrometer (EDX Genesis 4000). Before SEM micrograph acquisition, the materials were sputter coated with a tinny film of Au/Pd under a nitrogen stream at reduced pressure.

### TPC quantification with ZIF-8@paper device

The quantification of TPC was performed by loading 50 µL of standard/sample onto the top layer sampling hole (T in Fig. 1), placed over the detection layer. This volume exceeded the hydrophilic layer capacities, thus, some standard/sample volume would come out through the bottom layer hole (B in Fig. 1), but the phenolic compounds stayed retained in the ZIF-8 MOF at the FC layer (L1 in Fig. 1) since the MOF amount (*ca.* 150 µg) was enough to ensure the analyte (*ca.* 0.75 µg) retention.

After 20 min, at room temperature (20–25 °C), the volume was completely absorbed and the blue color developed. Then, the images (.jpg format) were acquired with a scanner (Epson) and processed using the free software ImageJ (red filter after RGB stacking).

A pseudo absorbance was calculated as absorbance signal using the expression: $\mathrm{A}={\mathrm{log}}_{10}\left(\frac{{\mathrm{I}}_{\mathrm{B}}}{{\mathrm{I}}_{\mathrm{S}}}\right)$, where I_B_ is the intensity of the blank signal attained loading MQW and I_S_ is the intensity signal attained from loading standard and/or samples. The intensity counts were always measured in circular segments (48 × 48 pixels) and the signal was established using 6 replicates per measurement (blank/standard) removing potential outliers, if necessary.

### Sample analysis with the developed ZIF-8@paper device

Several fruit samples (details in Table S2) were purchased in a local store and stored at − 20 °C until their use. The sample pre-treatment consisted of cutting, crushing, and filtering; the latter was made using gauze and a 25 mL plastic syringe, to remove solid particles. This simple sampling process can be carried out by non-trained personnel.

The filtered sample was collected into a flask and diluted (Table S2) prior to loading in the developed µPAD. The TPC quantification was obtained by absorbance interpolation in calibration curves obtained with oenotan standards. To minimize the sample color interference in the signal registrations, a “sample blank” was included and its value was subtracted to the signal reading. The “sample blank” reading was obtained in detection units prepared without adding FC reagent in the preparation process. However, in most samples, the necessary dilution of the sample pre-treatment was enough to minimize the contribution of the sample’s intrinsic color.

### Comparison method—Folin-Ciocalteu Index

To assess the accuracy of the developed ZIF-8@paper device, the µPAD results obtained for TPC quantification in fruit samples were compared with the reference method from International Methods of Wine and Must Analysis (OIV reference OIV-MA-AS2-10) using the Folin-Ciocalteu Index [23], since there is not a recommended method to establish the TPC content in fruit samples.

The method consisted of adding 5 mL of MQW to a test tube containing 0.2 mL of diluted fruit extracts/oenotan standard and thoroughly mixed (MQW in the case of blank). Then, 1 mL of commercial FC reagent (2 M) was added, the mixture was vortexed for 30 s, and afterward, in order to achieve alkali conditions (best chromophore adsorption), 4 mL of anhydrous Na_2_CO_3_ (20%, m/v) were added and the volume was adjusted to 20 mL in a volumetric flask with MQW. After 30 min of reaction time, the absorbance measurement was carried out after at 750 nm in Helios Gamma UV–Vis spectrophotometer (Thermo Scientific, Massachusetts, EUA). against an MQW blank. The expected absorbance for fruit samples, namely blueberry and strawberry, should be around 0.3, consequently, dilutions should be made.

## Results and discussion

The analysis of complex samples with paper-based devices is highly challenging due to the use of reduced sample volumes (≈ 20 µL) and potential matrix interferences. In this context, targeting the determination of total phenolic compounds content (TPC) in fruits, our previous work [26] was revisited to attain a performance enhancement and overcoming the expected challenges of solid samples. The same colorimetric reaction with Folin-Ciocalteu (FC) reagent was used, and the concentration of the FC reagent, the concentration of carbonate buffer solution, the type of filter paper, and the diameter of the discs were adopted from that work [26]. The new device should tackle the challenge of aiming important dietary fruits of phenolic compounds source enabling the direct TPC analysis with no need for laborious samples pre-treatments.

### Preliminary studies—choice of MOF particles

In order to improve the sensitivity, and decrease the LOD, a strategy to enable the use of a higher sample volume had to be defined. In this context, the introduction of MOFs in a µPAD with sensing purposes was considered since as we mentioned above, MOFs have fascinating properties to retain analytes. Therefore, a preliminary study was performed in order to establish which material was suitable for PC capture (Table S3 and Fig. S1) and the results showed that the retention was higher when ZIF-8 was used as an adsorbent. In this study, we have selected MOF from different families in order to study as many different properties as possible. In this sense, it is worth mentioning that MOFs with dark tonalities or blue color such as MIL-101 (Fe, Cr) or HKUST-1, respectively, would lead to a noticeable color interference in the µPAD detection, and they were consequently discarded, even if they have been reported as good adsorbents for PC [27, 28]. Taking into account the ZIF-8 retention [29, 30] and its white color (the same as paper), this MOF was selected for further studies.

### Incorporation of MOF ZIF-8 on paper

The incorporation of the ZIF-8 on paper was studied taking into account several aspects such as MOF suspension, loading order, and paper layer direction.

The loading order was assessed by comparing the calibration curves obtained when loading the MOF suspension before and after the FC reagent on the W541 filter paper disc. Loading the MOF suspension before FC reagent led to a sensitivity decrease from 7.8 to 6.4 L g^−1^ (~ 20%), consequently, the MOF suspension was loaded after FC reagent impregnation.

Then, it was studied the best MOF suspension to coat the cellulosic wall and the MOF dispersed in a methanolic solution was compared to the use of a sealing agent namely chitosan [31] and starch [32]. A slight loss of signal response was observed when using the sealing agents compared to direct deposited MOF (data not shown).

Hence, taking into account the signal loss together with the reduction of assembly time, the direct deposition (drop-casting method) of MOF obtained by the ZIF-8 suspension in MeOH (10 g L^−1^) was adopted for simplicity and convenience for the following studies.

Once established the MOF suspension and loading order, ZIF-8 MOF was to be set at the first hydrophilic layer aligned with the sample insertion hole on the top sheet of the laminating pouch (layer L1 and T in Fig. 1).

In order to prevent any MOF loss through the sampling hole, two configurations were tested: (i) having the hydrophilic first layer face down, and (ii) having an extra empty layer (qualitative Whatman® paper W1) between the top laminating pouch sheet and the reagent layer. The latter option hindered the color development and increased the assembly time since at least 3 discs per detection unit was needed; therefore, it was discarded. Concerning to the first approach, the sensitivity was similar to that obtained by having the reagent face up; therefore, both strategies could be employed.

From these preliminary experiments, the resulting final assembly incorporated the ZIF-8 MOF in the top layer via drop casting of a ZIF-8 suspension in MeOH (5 µL of 10 g L^−1^ in MeOH in triplicate) (Fig. 1A).

### Characterization of ZIF-8@paper

**Fig. 2:**
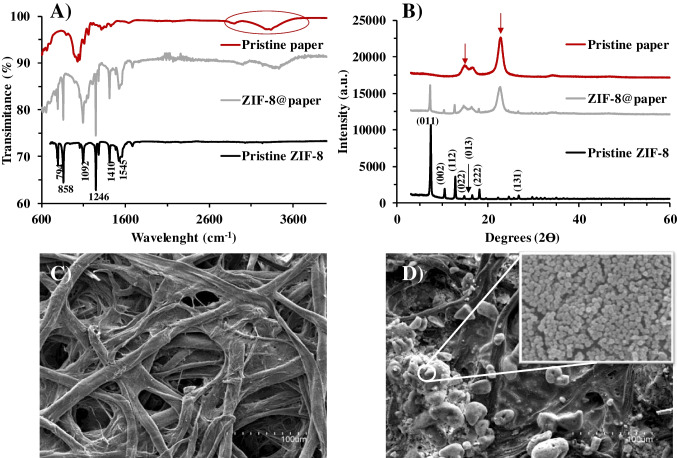
Characterization studies of ZIF-8@paper. **A**, FT-IR spectra, **B**, p-XRD profiles, **C** and **D**, SEM micrographs of the pristine and hybridized paper, respectively

An extensive characterization study of the final MOF-based cellulosic paper was done in order to (i) confirm the successful ZIF-8 synthesis, (ii) study its morphological and structural properties; and (iii) corroborate the introduction of ZIF-8 in the paper discs. For comparison purposes, controls of the as-synthesized ZIF-8 (black lines) and pristine paper (garnet lines) were included in these studies. Regarding the FT-IR spectra, Fig. 2A shows the characteristic peaks from ZIF-8 in both pristine and ZIF-8@paper (1545, 1410, 1246, 1092, 858, 794 cm^−1^).

In general, the peaks with significant transmittance in the region < 800 cm^−1^ are attributed to the out-of-plane imidazole ring bending, whereas those found in the region comprised between 825 and 1350 cm^−1^ can be associated with in-plane bending [33, 34]. The peak centered around 1545 cm^−1^ can be assigned to the C = N stretch modes and the broad band located at 3000–3800 cm^−1^ can be related to hydroxyl functional groups and hydrogen bonds from the cellulose [35].

On the other hand, p-XRD analysis was used to confirm the crystallinity of the framework as shown in Fig. 2B. The most representative MOF peaks corresponding to (001), (002), (112), (022), (013), (222), and (131) are matching with those ones reported elsewhere [24, 36], which suggests that the typical sodalite structure of ZIF-8 is well-formed. Some of these typical peaks can be also seen in the ZIF-8@paper, but with lower intensities due to the contribution of paper (characteristic bands at 2θ = 16° and 23°in red line). This also confirms the presence of MOF in the final ZIF-8@paper device with unaltered crystallinity/integrity. Besides, the morphological exploration was performed by SEM analysis. Indeed, Fig. 2 shows the micrographs of the cellulosic paper before (C) and after (D) MOF impregnation. As it can be seen, the surface was completely decorated with typical polyhedral MOF nanocrystals [37, 38], with particle sizes ranging from *ca.* 50 to 73 nm (*n* = 50), which tend to form aggregates (Fig. 2D). Additionally, EDX and mapping analysis of the bare paper and the ZIF-8@paper were performed. As depicted in Figure S2 and Table S4, the typical Zn bands were present in the EDX spectra and a Zn content in the ZIF-8@paper went up to 4 wt% and also an increase of the N content. Other characterization studies such as electron transmission microscopy, zeta potential of the ZIF-8 surface varying the pH and adsorption/desorption N_2_ isotherms (surface area *ca.* 1450 m^2^ g^−1^) can be found in the literature to go deeper into this section [24].

### ZIF-8@paper µPAD method optimization

The optimization of TPC determination with the developed ZIF-8@paper µPAD method was made using gallic acid standards as the model compound. The layer arrangement was set using FC reagent (0.5 M) in the first layer (T) and the carbonate solution in the second layer (B). After the design, the followed approach was to increase the sample/standard volume, thus, different sample volumes were tested, ranging from 25 to 75 µL.

It was expected that the ZIF-8@paper be more efficient in minimizing dispersion, but it is first necessary for a physical reconfiguration since those volumes exceed the hydrophilic layer capacities. Consequently, an additional hole was added in the bottom layer, aligned with the sample hole (5 mm) in the top layer, thus allowing the extra sample drains out. The same size was used for both holes and 5 mm was set as the highest diameter possible for the top layer considering the paper disc of the reagent layer has 6.3 mm. The conditions of these studies and the results obtained in terms of regression equation were summarized in Table S5.

The results showed a significant increase from 25 to 50 µL of standard (38%) with increasing sample/standard volume; however, with 75 µL, there were spills from the sample introduction hole, and it took over 30 min to absorb the entire volume, which compromised the calibration curve slope. Therefore, 50 µL was selected as an optimum standard/sample volume.

**Fig. 3:**
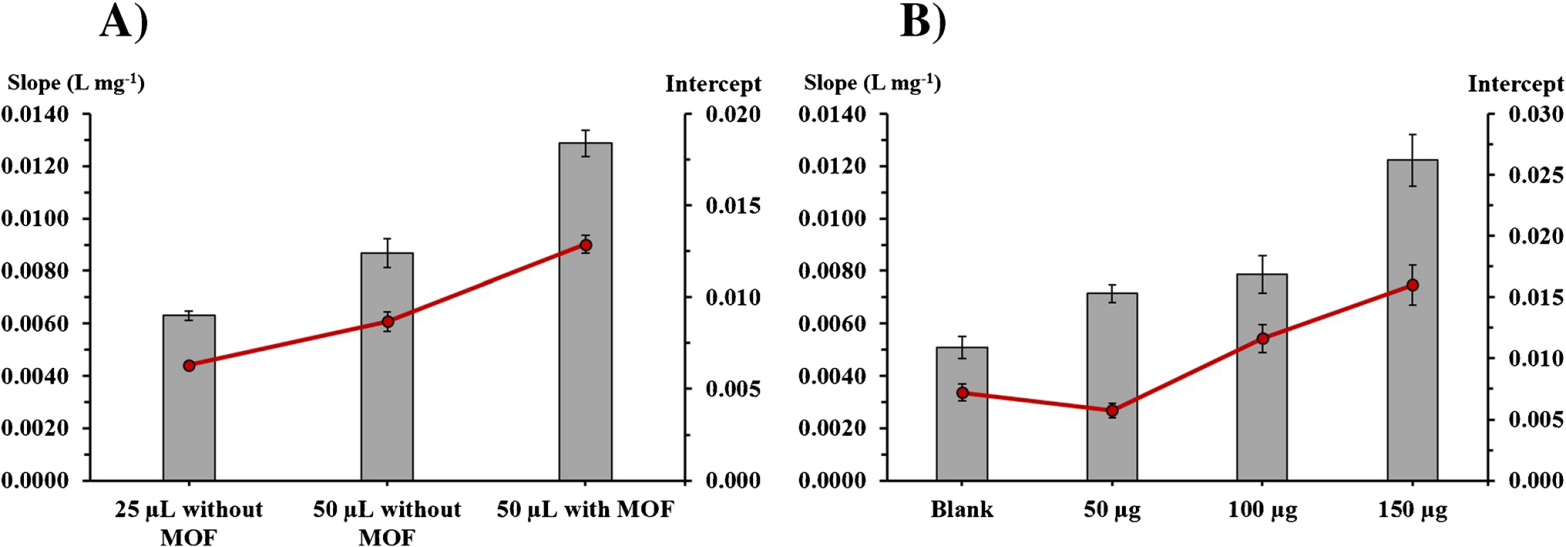
Sensitivity (slope) and interception values when varying sample volume (**A**) and MOF amount (**B**). Exp. conditions: **A**, 2 holes, W541 top layer 3/8″, 300 µg of ZIF-8; **B**, 2 holes, W541 top layer 1/4″, sample volume 50 µL

Then, calibration curves with different sample/standard volumes and MOF amount were compared (Fig. 3). As stated before, when the volume increased from 25 to 50 µL, a sensitivity enhancement was observed (even without MOF), but using 50 µL with the developed ZIF-8@paper µPAD results in a further increase of about 50% (Fig. 3A).

The impact of the MOF amount in the calibration curve was also studied and calibration curves were established for MOF dosage from 50 to 200 µg. The increase of the MOF amount resulted in an increase of the calibration curve slope; but with 200 µg of MOF, an interference in the signal was observed (higher intercepts) and, in some cases, a loss of linearity of calibration plot between A and (GA) was found (Fig. 3B). Therefore, 150 µg was set for further studies.

### Stability studies—developed colour and storage

**Fig. 4:**
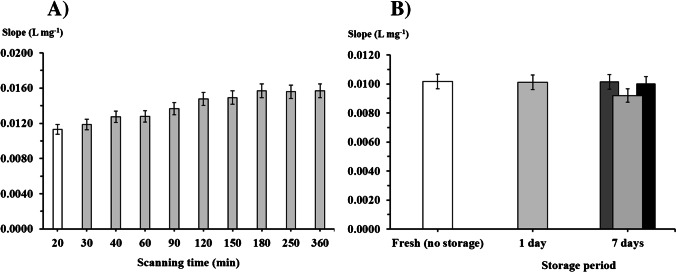
Stability studies of: **A**, the formed colored product, with different scanning times (light grey bars) after sample insertion compared to the chosen 20 min (white bar); **B**, comparing a freshly prepared device (white bar) with different storage periods and conditions, kept in vacuum (grey bars) at room temperature (light grey bar) and in the freezer (dark grey bar) and without vacuum just in the freezer (black bar); the error bars represent 5% deviation

Once the method was optimized (Table S6), the stability of the developed µPAD method using ZIF-8 was evaluated not only in terms of formed color product, but also in the device storage stability (Fig. 4).

Assessing the stability of the color product formed enables to determine the time interval between sample insertion and scanning for signal detection. The selected 20 min represents the minimal waiting time, but it is important to assess what happens if there is a higher time interval (Fig. 4A). The blue chromophore development produced a signal enhancement in the first 2 h, as also reported elsewhere [23], but then it became constant for up to 6 h (overlapping of the 5% variation intervals). This value (6 h) is enough for performing the in-field analysis (sample loading) and the posterior µPAD reading in the laboratory (data treatment).

The storage stability was also studied to establish how long the device could be kept after assembly and before its use. A couple of storage periods, 1 and 7 days, and conditions, in vacuum, room temperature, and freezer were evaluated (Fig. 4B). When the devices were kept at room temperature without vacuum, it was not possible to obtain a calibration curve, consequently, without vacuum, only the device kept in the freezer was considered (black bar in Fig. 4B). The devices were stable (overlapping of the 5% variation of the slope) when kept in vacuum for 1 day at room temperature, but not for 7 days (grey bars Fig. 4B). When the devices were kept in the freezer, they were stable with or without vacuum (overlapping of the 5% variation of the slope). Taking into account these results, the lifetime is at least 2 weeks in the freezer.

### Features of the developed ZIF-8@paper µPAD

Regarding the analytical features of the developed ZIF-8@paper µPAD, a summary is presented in Table 1, including linear range, limit of detection (LOD) and limit of quantification (LOQ), precision, and stability.

**Table 1:** Analytical quality parameters of ZIF-8@paper cards for the quantification of TPC using gallic acid (G.A.) as reference phenolic compound

Analytical parameter	Value
Linear range	1.2–30 mg L^−1^
Calibration equation (*n* = 5),	A = (1.29 ± 0.16)·10^−2^·(G.A.) + (9 ± 2)·10^−3^
A = slope ± SD (G.A.) mg L^−1^ + intercept ± SD	*R*^2^ = 0.999 ± 0.001
LOD	0.36 mg L^−1^
LOQ	1.2 mg L^−1^
Precision intra-device (*n* = 6)	RSD = 9.0% (for 20 mg L^−1^)
Precision intra-day (*n* = 4)	RSD = 7.9%
Precision inter-day (*n* = 3)	RSD = 7.6%
Colour stability after sample addition	Up to 6 h
Storage stability^a^	At least 2 weeks
Consumption/µPAD	5 μL of 0.5 M Folin Ciocalteu (F.C.)
	72 mg carbonate
	0.15 mg MOF
	50 µL sample

^a^Storage in vacuum, darkness and − 20 °C

*LOD* limit of detection, *LOQ* limit of quantification, *SD* standard deviation, *RSD* relative standard deviation

The LOD and LOQ were calculated following IUPAC recommendations [39] as the concentration corresponding to three and ten times the standard deviation of the intercept (*n* = 5), respectively. The precision of the µPAD card was assessed by calculating the relative standard deviation (RSD) of six signal readings for a 20 mg L^−1^ of gallic acid standard. The precision of the method was evaluated by calculating the intra-day and inter-day RSD of the calibration curve slope (sensitivity). The calibration curves on the same day were made with the same batch reagents and the calibration curves of different days were made with a new batch of reagents. The consumption was also calculated based upon the volumes used for loading one µPAD.

### Application to fruit samples

The feasibility and accuracy of the developed method were established by applying the µPAD to TPC quantification in complex matrices, namely fruits, and comparing the results to those obtained with a reference procedure. These samples commonly contain about 85% of water and 13% of carbohydrates. In a previous work, developed for TPC in wines, it has been proved that contents up to 20 g L^−1^ of glucose (as representative monosaccharide) did not interfere in the determination [26]. Therefore, no significant interferences were expected and the samples were directly analyzed.

Thus, the fruit sample was prepared as mentioned (in the “Sample analysis with the developed ZIF-8@paper device” section) with no additional pre-treatment steps. A variety of fruit samples (#7) were selected with expected differences in TPC content: apple, blueberries, lemon, grapes, pear, pineapple, and strawberries (Table S2).

The direct TPC quantification (by interpolation in a calibration curve) produced better results using a tannin standard (oenotan) than the model compound gallic acid (Figure S3). This can be explained by the different molecular size between gallic acid and tannins, which results in the different distribution in the paper. Then, for the TPC determination in the fruit samples, the calibration curve was established with oenotan standards (Figure S3B). The results obtained from the developed µPAD method were compared to the OIV reference protocol, which was also established with oenotan standards (Table 2).

**Table 2:** Determination of TPC in different fruits using both the developed µPAD method (µPAD) and the recommended procedure by the International Organisation of Vine and Wine (OIV)

Sample	OIV^a^		µPAD^b^		Relative error (%)
	(TPC) ± SD, g/L		(TPC) ± SD, g/L		
Apple	0.697	± 0.002	0.699	± 0.067	0.2
Blueberry	1.09	± 0.04	1.04	± 0.06	− 4.8
Grapes	0.965	± 0.020	0.919	± 0.022	− 4.8
Lemon	0.515	± 0.037	0.495	± 0.056	− 3.8
Pear	0.344	± 0.020	0.364	± 0.002	5.8
Pineaple_stem	1.02	± 0.03	1.00	± 0.07	− 1.4
Pineaple_peel	1.06	± 0.03	1.05	± 0.16	− 1.5
Strawberry	1.95	± 0.10	2.07	± 0.13	6.4

^a^*n* = 3

^b^*n* = 5

*SD* standard deviation

The results proved to be comparable (relative error < 6.4%) and a linear relationship was established between the two sets of results (Figure S4) with the equation [TPC]_µPAD_ = 1.07 (± 0.09) [TPC]_OIV_ – 63.1 (± 98.0), where the values in brackets represent 95% confidence interval. There are no statistical differences between the two sets of results since the slope is not statistically different from 1 and the intercept is not statistically different from 0.

## Conclusions

In this study, a user-friendly and portable µPAD, combining MOF and cellulosic paper, has been developed. The method was successfully applied to quantify the TPC in several fruit samples. The principle of the reaction is based upon the FC Index with a blue color development of the resulting phosphotungsticphosphomolybdenum complex in presence of reducing agents (phenolic compounds) at alkali medium. Some physico-chemical parameters affecting the µPAD performance were optimized and the quality parameters of the method were established. Despite the modest linear range reached from 1.2–30 mg L^*−*1^, high sensitivity and low LOD (0.36 mg L^−1^) were achieved, which could be very interesting when complex food samples are being analyzed. For instance, a high dilution factor can be done in order to avoid the sample matrix.

Although the upper linear range decreased from 50 to 30 mg L^−1^ (with determination coefficient higher than 0.99) with our previously developed µPAD method [26] (being a disadvantage), the lower linear range was enhanced from 4.1 to 1.2 mg L^−1^ (approx. threefold referred to LOQ value) as well as a twofold increase of the slope (from 0.0066 to 0.0129 L mg^−1^).

The developed method has been compared with other recently reported paper-based technologies in order to corroborate the suitability of its application (Table 3). For instance, our device not only is performed by simple stationery material but also does not require the heating of wax [40, 41] neither toxic organic inks [42]. Surprisingly, Vaher et al. [43] did not introduce hydrophobic barriers between the different reaction zones, which can easily lead to cross-contamination. Furthermore, our cards present 24 reaction zones per device, allowing a high throughput low cost per device (< 50 cents).

**Table 3:** Comparison of the most recent contributions regarding optical paper-based sensors for phenolic content determination

Device	Hydrophobic barrier	Sample matrix	Sample volume (µL)	LOD (mg L^−1^)	Precision (RSD, %)	Storage stability (conditions)	Ref
3-holes lateral-flow µPAD	Pen ink	Tea, wine, and beer	2	2	N.R	N.R	[42]
4-holes lateral-flow µPAD	Wax printing	Water	50	4.5	< 8	2 months (r.t.)	[40]
Paper microzone plates	None	Wines	2	40	< 16	N.R	[43]
4-holes lateral-flow µPAD	Wax printing	Tea, wine, and fruit juices	10	85	< 10	1 month (4 °C)	[41]
24-holes vertical-flow µPAD	Laminating pouch	Wines	25	1.2	< 9	1 month (vacuum, r.t.)	[26]
24-holes vertical-flow µPAD	Laminating pouch	Fruits	50	0.36	< 8	2 weeks (− 20 °C)	This work

*N.R.* not reported

On the other hand, our approach presents the lowest LOD for the detection of TPC, probably due to the presence of ZIF-8 and the high volume loaded, which is really important when complex samples are analyzed by diluting the sample matrix. As it can be seen in the table, only the microzone plates approach presents a distinctive higher RSD (< 16%) [43], whereas the other reported precisions are in the range comprised between 8 and 10% [26, 40–42]. In terms of storage stability, although some authors have not stated values [42, 43], whereas the present method presents lower stabilities than the other reported devices [26, 40, 41]. In any case, 2 weeks of µPAD stability is more than enough to assure its shipping to low-resource places, which has a synergistic effect with the 6 h color stability after the sampling.

Furthermore, comparing our device with other reported work for TPC in fruit juices, the LOD is around 170 times lower (85 mg L^**−**1^ [41]), which can be highly advantageous when high dilution factors are employed to minimize matrix interference. It is also worth mentioning that all the possible reductors present in the sample could react with FC reagent, thus overestimating the TPC in real samples. In any case, the described method is suitable for TPC quantification in fruit samples without any additional sample pre-treatment besides simple smashing and diluting as it proved to match the values obtained with the reference method. With a cost lower than 50 cents per unit, this smart approach represents an alternative to sophisticated instrumentation for in-field applications, which opens lines in the new MOF-based methodologies for rapid and environmentally friendly screening outlines.

## Supplementary Information

Below is the link to the electronic supplementary material.

Supplementary file1 (PDF 503 KB)

## Data Availability

Data of this reasearch is included in the manuscript and supplementary material, any other data can be provided upon request.
