# Supplementary material for: Combining microfluidic paper-based platform and metal–organic frameworks in a single device for phenolic content assessment in fruits
Source: Mikrochim Acta. 2023 Mar 10;190(4):126. doi: 10.1007/s00604-023-05702-5 (PMC10006271; doi:10.1007/s00604-023-05702-5)
Supplement: Supplementary file 1 — Supplementary file1 (PDF 503 KB) [file 604_2023_5702_MOESM1_ESM.pdf]

ELECTRONIC SUPPLEMENTARY MATERIAL

Combining microfluidic paper-based platform and metal-organic frameworks in a single device for phenolic content assessment in fruits

H. Martínez-Pérez-Cejuela[1,2], Raquel B. R. Mesquita[2*], E.F. Simó-Alfonso[1], J.M. Herrero-Martínez[1*], António O. S. S. Rangel[2]

[1]*Department of Analytical Chemistry, University of Valencia, Dr Moliner 50, 46100, Burjassot, Valencia, Spain*

[2]*Universidade Católica Portuguesa, CBQF - Centro de Biotecnologia e Química Fina – Laboratório Associado, Escola Superior de Biotecnologia, Rua Diogo Botelho 1327, 4169-005 Porto, Portugal*

Corresponding authors:
Raquel B. R. Mesquita, Tel.: +351 225 580 046 (rmesquita@ucp.pt)
José Manuel Herrero Martínez Tel.: +34 963 544 062 (jmherrer@uv.es)

Table S1. Properties of the Whatman® filter papers used as top and bottom layers in the developed µPAD design.

Whatman® type	Paper nature	Pore size (µm)	Thickness (mm)
W3	Qualitative	6	0.39
W541	Hardened ashless	20-25	0.16

Table S2. Fruit samples analyzed in this work.

Sample	Origin	Variety/type	Dilution factor
Apple	Portugal	Fuji	1/20
Blueberry	Spain	-	1/50
Grapes	Portugal	Touriga nacional	1/40
Lemon	Portugal	Siciliano	1/20
Pear	Portugal	Packham	1/20
Pineapple	Açores - Portugal	Stem	1/50
		Peel	1/50
Strawberry	Spain	Sabrina	1/100

Table S3. Conditions of preliminary dispersive-SPE study for MOF selection using gallic acid as reference phenolic compound and measuring the remaining fraction (OIV [1]).

MOFs	Amount	Time of contact	Analyte	Agitation	Analysis
ZIF-8, MIL-101, UiO-66	10 mg	15 min	2 mL of 25 mg L^{-1} of Gallic acid	Vortex-assisted	Folin Index

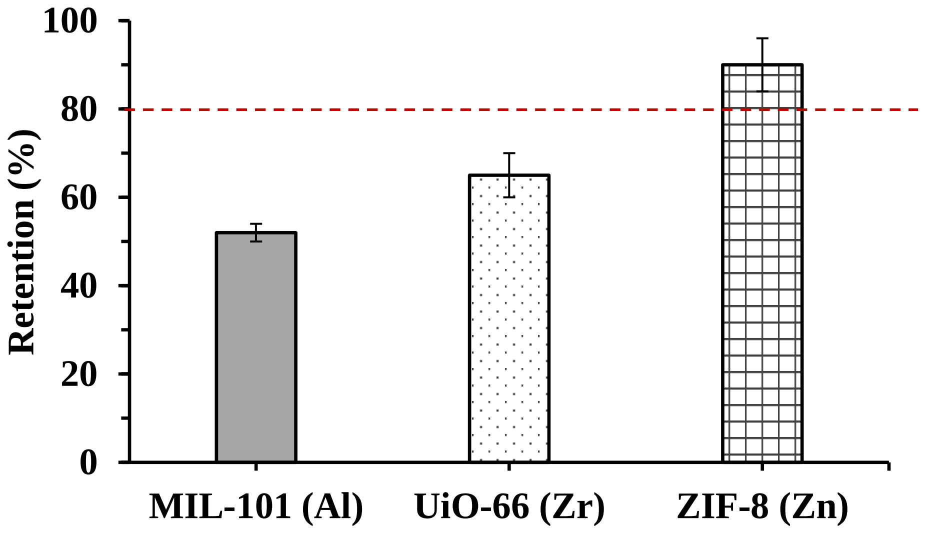

Figure S1. Retention values of different MOFs from preliminary dispersive-SPE study.

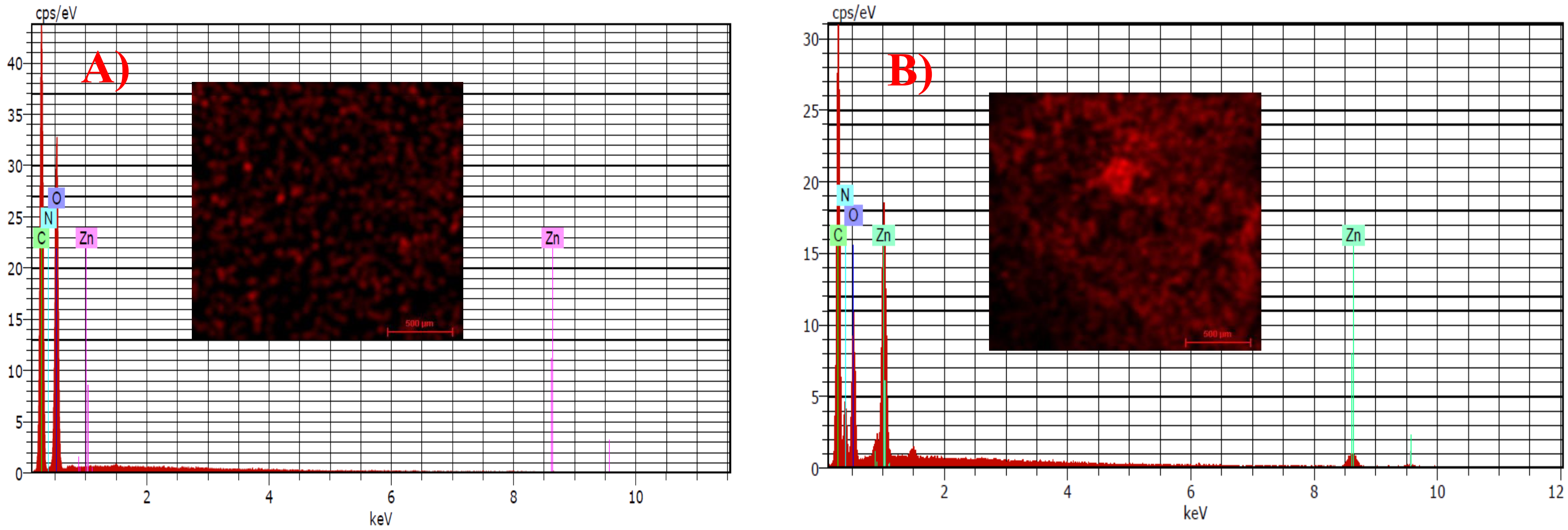

Figure S2. EDX and mapping analysis of (a) bare cellulose paper and (b) ZIF-8@paper.

Table S4. Composition of bare paper and ZIF-8@paper using EDX analysis.

Sample	C (wt. %)	N (wt. %)	O (wt. %)	Zn (wt. %)
Bare paper	45.4	2.5	52.1	0
ZIF-8@paper	41.5	23.3	31.3	3.9

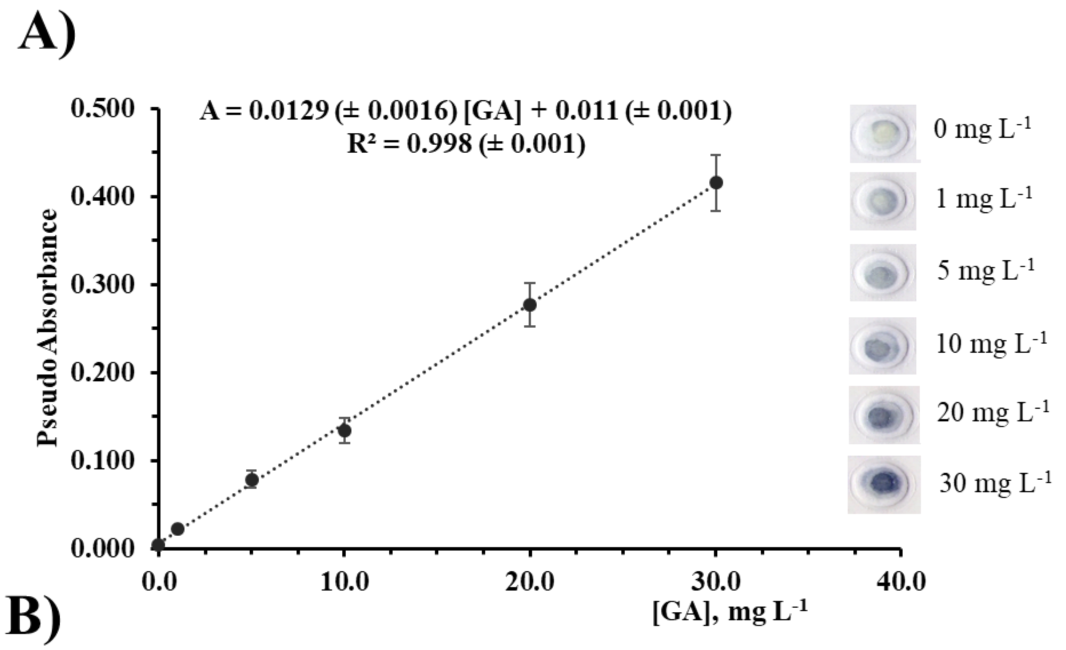

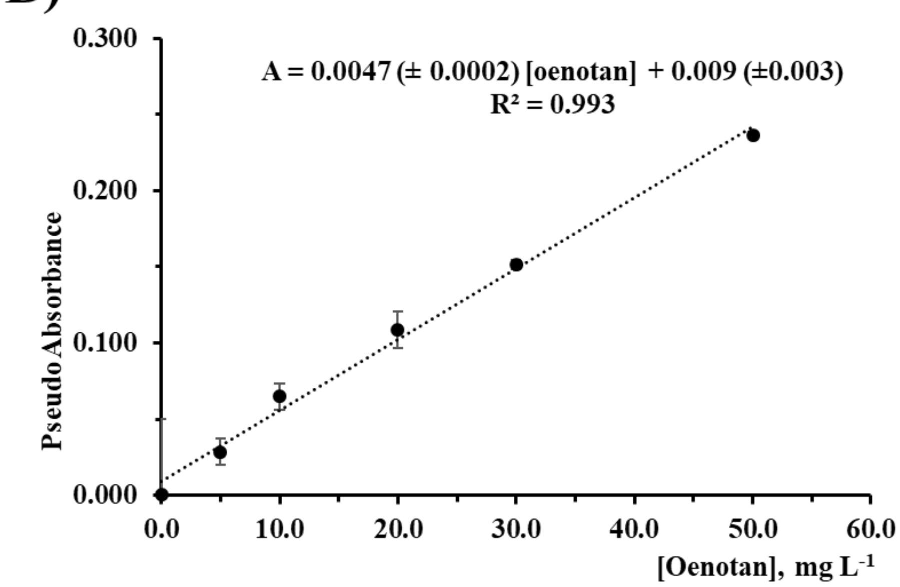

Figure S3. Calibration plots for the TPC quantification; A) with Gallic acid (0–30 mg L^{-1}) and representation of colour response after 20 min of incubation time. B) with oenotan (0–50 mg L^{-1}); Error bars represent the standard deviation (n = 4). Experimental conditions are given in Sections 2.3 and 2.5.

Table S5. Influence of the sample volume influence on the calibration plot slope using gallic acid standards in the range 10 – 30 mg/L; design conditions: top layer paper disc W451 (3/8") with 10 µL of F.C. 0.5 M and bottom layer paper W3 (3/8") with 20 µL N_2CO_3 15% (m/v).

**Sample volume (µL)**	**Holes**	**Sensitivity (Slope L mg^{-1})**
25	1 (top)	Slope = 0.0063
50	2 (top/bottom)	Slope = 0.0087
75	2(top/bottom)	Slope = 0.0027

Table S6. Optimized experimental parameters for the quantification of TPC in fruit samples with the developed MOF@µPAD method.

**Parameters**	**Optimal value**
Top layer, type (pore size, thickness, diameter)	W541 (20-25 µm, 0.16 µm, 1/4'')
F-C reagent (M)	0.5
MOF amount (µg)	150
Bottom layer, type (pore size, thickness, diameter)	W3 (6 µm, 0.39 µm, 3/8'')
Na_2CO_3 (% m/v)	15
Sample volume (µL)	50

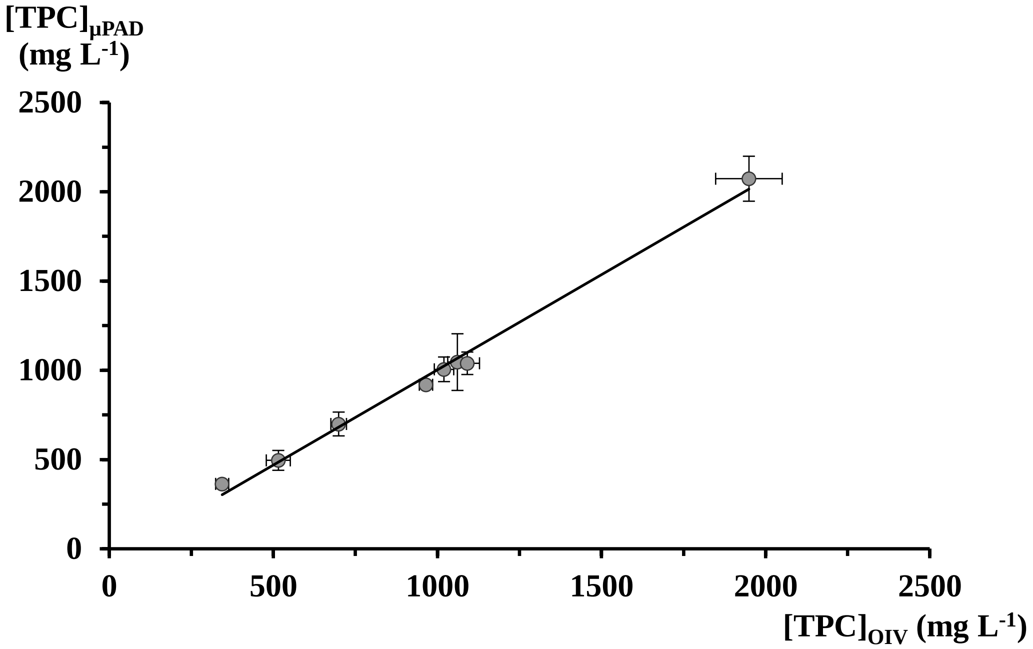

Figure S4. Pearson plot of TPC quantification using the developed ZIF-8@paper devices ($[TPC]_{\mu PAD}$) and the spectrophotometric reference method ($[TPC]_{OIV}$); error bars mean the standard deviation (n = 6).

References

1. Singleton VL, Rossi JA (1965) Colorimetry of Total Phenolics with Phosphomolybdic-Phosphotungstic Acid Reagents. Am J Enol Vitic 16:144–158
